# What can climate services learn from the broader services literature?

**DOI:** 10.1007/s10584-019-02388-8

**Published:** 2019-02-18

**Authors:** Meghan Alexander, Suraje Dessai

**Affiliations:** 1 School of Earth & Ocean Sciences, Cardiff University, Cardiff, CF10 3AT UK; 2 Sustainability Research Institute and ESRC Centre for Climate Change Economics and Policy, School of Earth & Environment, University of Leeds, Leeds, LS2 9JT UK

## Abstract

Climate services seek the timely production and delivery of useful climate information to decision-makers, yet there continues to be a reported ‘usability gap’. To address this, many have advocated the coproduction of climate services between knowledge producers, providers and users, with a tendency to focus on tailoring information products to user needs, with less attention towards the service environment itself. In service management and service marketing fields, this is referred to as the ‘servicescape’ and is shown to influence behavioural intention, value creation and perceived service quality. In an effort to facilitate cross-disciplinary learning, this research asks whether climate services can learn from other service-based research in public administration/management, service management and service marketing. Performing a semi-deductive literature review, this perspective article examines themes of coproduction and servicescapes, and identifies relevant topics for future climate services research around the added value of service-dominant logic, the subjective experience of users’ interaction with servicescapes, and empowerment of users as co-producers of value. This is an important first step in promoting further cross-disciplinary learning to advance both scholarship and operational delivery of climate services.

## Introduction

The importance of climate services in society has long been recognised (Hecht 1984), yet the concept has more recently gained momentum in research, industry and institutional initiatives (WMO 2014; Vaughan and Dessai 2014; European Commission 2015). Climate services embody the production, translation and transfer of climate research into usable information for climate-related decision-making (Vaughan et al. 2018). Although dominated by national meteorological services, climate services exist across the public-private spectrum and support a range of climate-sensitive sectors (Brasseur and Gallardo 2016; Lourenço et al. 2016).

However, researchers have continued to observe a ‘usability gap’ (Lemos et al. 2012). This has been attributed to the tendency towards science-driven (as opposed to demand-driven) climate services (Lourenço et al. 2016), resulting in calls for actionable information and tailored services (Dilling and Lemos 2011; Kirchhoff et al. 2013). Barriers to the uptake of climate services are wide-ranging, relating to communication, accessibility, relevance, usability and capacity (Bruno Soares and Dessai 2015). To overcome these, many have called for improved knowledge exchange and collaboration between knowledge producers, providers and users, otherwise referred to as coproduction (Meadow et al. 2015; Briley et al. 2015; Bremer and Meisch 2017). To a lesser extent, some researchers have also involved users in the design of service platforms and examined their interaction with the (virtual) service environment in an effort to enhance the usability of climate services (Hewitson et al. 2017; Christel et al. 2018).

Although climate services represent an emerging field, the study of services more broadly is not new and maintains a long legacy in public administration, service management and marketing disciplines in particular. However, although the language of business and corporate services has permeated climate services (Webber and Donner 2017), few authors have sought to examine the parallels and potential transference of knowledge across different service contexts. Of these few, Troccoli (2018) argues that climate services are not unique from other services, although arguably suffer from higher levels of risk (for instance, in terms of the reliability and accuracy of climate information). Ultimately, a service aims to satisfy its users, ‘by extracting the highest value from, in our case, weather and climate information, for the specific application at hand’ (Troccoli 2018, p. 14). Every service comprises providers, beneficiaries, goods/products and service delivery mechanisms and, whether delivered through the public policy process to provide public goods, or initiated through private investment or market regulation, the core definition remains the same (Troccoli, ibid). Although the beneficiaries of services are referred to under various guises as clients, users, customers or citizens, these terms can arguably be thought of as synonymous in the sense that all services must ultimately address the requirements of these groups. This makes the transference of lessons from other service-based research to climate services possible.

This perspective article broadens the conversation around climate services by examining the broader services literature. Given the considerable breadth of service-based research, we focus on the themes of (i) *coproduction*, which has steadily established itself in climate services research (Lemos et al. 2012; Vaughan et al. 2018), and (ii) the notion of *servicescapes*. The latter theme derived from our early reading of service management and service marketing literature, and addresses the influence of interactions between service users, providers and service environments upon users’ intentions, value creation and perceived service quality (Reimer and Kuehn 2005; Mari and Poggesi 2013). This research area is noticeably lacking from current climate services, but is arguably implicit in research focused on the design of user-interfaces (e.g. Christel et al. 2018) and users’ experiences of climate information websites (Hewitson et al. 2017), suggesting that servicescapes are of interest to climate services scholarship. These themes were selected as starting points only in an effort to demonstrate the potential for cross-disciplinary learning.

## Methodology

An extensive repository of public-private services research exists within public administration/management, service management and service marketing. An initial search in Scopus for ‘service*’, ‘public’ OR ‘private’ (limited to title, abstract, author-identified key words, articles, reviews and articles in press in journals published in English, and excluding certain subject areas^1^) reveals an initial sample of 174,543 articles. Using this as a base sample, the search was refined to focus on *coproduction* and *servicescapes*, as useful starting points for cross-disciplinary learning.

Adopting a semi-deductive approach, this research performed a high-level review utilising key search terms (Table 1). The research introduces climate services researchers to disciplinary insights that might otherwise have remained elusive; therefore, we purposively selected articles that presented compelling concepts and interesting possibilities for climate services research. Whilst this is defensible in the context of the research’s aim, we acknowledge its subjectivity and recognise that others may have identified different articles and concepts. Therefore, this article should be regarded as a first step into these adjacent disciplines, from which future research and collaborations should be sought.

**Table 1:** Key search terms and number of articles identified in Scopus (derived from initial base search for public or private services, as outlined above)

	Coproduction search terms [‘co-prod*’ OR ‘coprod*’]	‘servicescape*’
Total number of articles	2283	722
Documents by country or territory (top 5)	UK, USA, Australia, Netherlands and Germany	USA, UK, Australia, South Korea and Taiwan
Documents by source (top 5)	*Public Management Review; Service Industries Journal*; *Ecosystem Services*; *Voluntas*; *Journal of Service Management*	*Journal of Services Marketing*; *International Journal of Hospitality Management*; *Journal of Retailing and Consumer Services*; *Journal of Service Research*; *International Journal of Contemporary Hospitality Management*.
Documents per year (general trend)	Increasing from 17 articles in 2002 to 362 articles in 2017	Increasing from 8 articles in 2002 to 95 articles in 2017

To assist purposive sampling, we performed a process of ‘abstract sifting’ to help sensitise ourselves to the language and subject matters of disparate disciplines, and the presence of compelling concepts. This is essentially a speed-reading process, using the title, abstract and author-identified key words, to sort and filter vast bodies of literature for purposive sampling. Snowball sampling was further employed to identify additional literature cited within the initial sample.

The remainder of this article focuses on the literature that we believe captures concepts and findings of interest to the climate services community. Some of these may also be applicable to weather services given its similarity to climate services (Troccoli 2018); however, as climate services are less well established, this constitutes our focus. It is necessary to remain critical of the extent to which lessons can be transferred across public-private divides, given their different remits (e.g. profit vs addressing social needs; Osbourne et al. 2012), but, as climate services span this spectrum, we include both. Finally, readers should be cognisant of the types of services and contextual settings represented in the sample, noting the bias towards developed nations (Table 1). It is not the intention to generalise the observations made in this article to climate services worldwide or to all types of climate services; instead, this article identifies relevant research avenues for climate services scholars to pursue through contextually situated research.

## Insights into the coproduction of services

Coproduction has been debated since the 1970s in relation to the role of citizens in service delivery, with the concept evolving through the disparate disciplines of public administration and public management (Ostrom and Ostrom 1977; Brudney and England 1983; Ostrom 1996; Alford 2014), and service management and service marketing (Vargo et al. 2008). There is a strong consensus that coproduction pertains to the voluntary and active interactions that take place between state/citizens or service providers/users, and the reciprocal use of each other’s assets, resources and contributions to achieve better outcomes in professionalised services (Verschuere et al. 2012; Bovaird et al. 2015). Looking at this repository of services literature, we identified several areas of interest for climate services scholarship, relating to goods and service-dominant logic, coproduction typologies, and users’ motivation to coproduce.

### Goods or service-dominant logic?

The relationship between services and coproduction varies between disciplines. Within public administration and new public management, coproduction has been typically framed as a voluntary component to delivering public services, whereby users are added into the process and invited to give their opinion on service improvement (Osborne and Strokosch 2013). This perspective on coproduction emerged from the ‘goods dominant logic’ of manufacturing management and research concerning the production and transaction of discrete goods. In public administration, this has arguably resulted in the treatment of “public services as ‘goods’ to be designed, planned and produced primarily by service professionals - but where service users can be invited into the process”, at the behest and control of professionals (Osborne and Strokosch 2013: 34). Consequently, coproduction has been typically treated as an optional component of service design and planning, external to service delivery. However, certain scholars have challenged the suitability of this premise for public services, which often involve more intangible service processes (Osbourne et al. 2012; Alford 2014).

In contrast, service management and marketing literature adopt a service-dominant logic, thereby shifting the emphasis to service delivery and casting coproduction as both integral and intrinsic component (Grönroos 2011). The production and consumption of many services are seen to take place simultaneously, such as restaurant dining or consultation with a solicitor for example (Osborne and Strokosch 2013). Therefore, the value is not simply determined by the quality of the product or good provided, but rather through provider-user interactions, the user’s expectations and their subjective experience, as well as the consumption experience whereby value is created in-use (Lusch and Vargo 2006; Ramaswamy 2011). From this standpoint, customers are regarded as co-creators of both the service experience and of value (Prahalad and Ramaswamy 2004). To enhance the value of the service therefore requires understanding of users’ expectations and requirements, careful management of service experiences and innovating the service environment (Nilsson and Ballantyne 2014). Although much of this research has been rooted in private sector services, others have argued for a ‘public service-dominant approach’ (Osbourne et al. 2012). Recognising that public services often involve both goods and service components, tensions between the two logics can arguably be overcome by either adopting a more holistic view on coproduction or dividing activities into service and goods components (Alford 2014).

This begs the question, how is coproduction positioned in climate services research and practice? The literature suggests that practice has tended towards a public administration perspective in the past, whereby coproduction has arguably been treated as an ‘add on’ component, if acknowledged at all, and controlled by service providers; indeed, only recently have science-driven climate services shifted towards user, demand-driven services (Lourenço et al. 2016). However, there continues to be traits of ‘goods-dominant logic’, whereby coproduction activities are arguably focused on the design of climate information products (e.g. Lemos et al. 2012) as opposed to service delivery and service experiences. This appears to be slowly changing as researchers examine relationships between providers, boundary organisations and service users (e.g. Briley et al. 2015), yet users are rarely referred to as co-creators of value, with few studies into service experiences (Hewitson et al. 2017). If climate services are founded on goods-dominant logic, there is a risk that users are treated as passive consumers involved in the discrete transaction of climate products, thus ignoring the processual nature of services and users’ role as co-producers (Osbourne et al. 2012). Therefore, we argue that climate services could benefit from embracing a more service-dominant culture that recognises the importance of users’ subjective experiences and empowers users as co-producers of value.

### Distinguishing different types of coproduction

Another interesting feature of the literature is the range of coproduction typologies that exist. Some examples are outlined in Table 2, alongside initial thoughts on how these might be evidenced in climate services. Whilst some of these examples focus on the role of citizens, Table 2 considers how these typologies might be adapted and applied to climate services to understand the relationship between service users and providers. To date, research into the coproduction of climate services has yet to examine how current practices ‘bolt onto’ such existing typologies or whether further nuances are required for the climate service context. This may prove challenging given the lack of explicit reporting on user engagement observed in climate services in practice. Indeed, in a study of 101 self-reported descriptions of climate services activities in 2012, Vaughan et al. (2018) note that more than half of providers did not mention specific users or user engagement in the development of the service. Nonetheless, interesting questions are raised about the forms of coproduction occurring in climate services and potential variations between different types or scales of operational services. The ability to differentiate between different coproduction types could help design coproduction initiatives linked to specific outcomes (Brandsen and Honingh 2016).

**Table 2:** Examples of coproduction typologies and implications for climate services

Author	Typology	Relating existing typologies of coproduction to climate services
Brandsen and Honingh (2016)	Identify 4 types of coproduction which are encouraged in public policy. These depend on (and combination thereof) (i) the extent to which citizens are involved in the design and/or implementation of professionally produced services and (ii) the proximity of the tasks that citizens perform to the core services of the organisation. *Complementary coproduction in service design and implementation*—pertains to citizens’ involvement in tasks that complement the core service (e.g. involvement of citizen volunteers in extracurricular activities). *Complementary coproduction in implementation*—citizens are actively involved in implementation but not the design of a complementary task (e.g. students assisting university open days). *Coproduction in the design and implementation of core services—*citizens are directly involved in the design and implementation of the core services provide to them (e.g. participative building projects). *Coproduction in the implementation of core services*—citizens are actively involved in the implementation but not the design of core services. This could be a deliberative decision, or, coproduction could be inherent to the successful implementation of core services (e.g. students are required to follow a defined curricular but their input is essential for effective learning).	Core tasks in climate services may include (i) the development, maintenance and management of the service platform (e.g. web-based interface) through which climate information products are obtained; (ii) strategic decisions pertaining to the design, scope and types of information products provided; (iii) the operational delivery of these; and (iv) the ongoing evaluation and user-consultation processes required to ensure the service is fit-for-purpose. The involvement of service users within the design and/or implementation of these tasks would qualify as coproduction in the core service. For instance, *coproduction in the design* of climate services may be evident in cases where users have been actively involved in strategic decisions about the scope and presentation of service platforms. *Coproduction in implementation* could be interpreted in 2 ways. Firstly, a deliberative decision may be made to involve users in a core aspect of service delivery for example, such as contributing climate information products (as is the case with citizen science). Alternatively, users may be inherently involved in the sense that their engagement in climate services is regarded as essential for its successful implementation. For example, the successful implementation of climate services more broadly could be thought of as dependent upon users’ willingness to learn about climate information products and meaningfully apply these to relevant decision-making contexts. The identification of so-called complementary tasks depends on what climate services providers have defined as core tasks. For instance, an example of *complementary coproduction* might include cases where providers have consulted users to elicit their feedback to improve the current service. In this example, the users themselves are not actively involved in designing or delivering the consultative exercise, but are no less important in helping to shape the service itself. This particular example could also arguably demonstrate a case of ‘coproduction in the implementation of a core service’ (assuming user-evaluation is seen as such), as the success of user-consultation in service evaluation (and subsequent service improvement) is inherently dependent on users’ input. In this instance, it would depend whether user-evaluation is regarded as a core or complementary task by the climate services provider.
Osborne and Strokosch (2013)	Seeking to develop a holistic model of coproduction, Osborne and Strokosch (2013) identify 3 coproduction types, each raising different questions and challenges. *Consumer co-production*—adopting a service management perspective, coproduction is seen as unavoidable component of service consumption; thus, coproduction is not an issue of choice or design, but instead requires the effective management of service delivery (and the relationships between service providers and users) at the operational level. Service quality and performance is dependent on individual users’ expectations and experiences, and their *empowerment* to actively negotiate their service experience and contribute to their own desired outcomes. The focus is on the individual’s consumption of the service at the operational scale (i.e. service delivery). *Participative co-production*—adopting a public administration perspective, a strategic decision is made to involve users to improve the design and planning of existing services, and how this might be improved for the future. This perspective is focused on user participation at the strategic scale of service planning. Biases may exist in who is included/excluded from participatory processes, but participation is intended to foster social inclusion in the decision-making process. *Enhanced co-production*—is seen as a distinct conceptual category, whereby the service user is regarded as a force for transformative change through which existing paradigms are challenged. This may be evidenced in user-led innovations, whereby users are co-creators in new service processes.	*Consumer co-production* in climate services is evident when attention is given to users’ expectations and experiences, and where these are used to help develop service delivery. Opportunities are created for users to actively influence their service experience and contribute to their own outcomes through their interaction with service providers and climate services platforms. Customised climate services are an example of this. Unlike consumer co-production, *participative co-production* requires the involvement of users in the strategic service planning. Workshops, focus groups and surveys may be used to engage service users in the design of future climate services (e.g. Copernicus Climate Change Service). There is no evidence of *enhanced coproduction* in the literature. Climate services are typically science-led and dictated by scientific and technological capabilities. True transformative change would need to challenge this existing paradigm and move beyond the current dominance of participative or consumer coproduction, which focus on tailoring existing services. Instead, ‘users’ of climate services would need to be regarded as ‘co-creators’ in some or all aspects of the climate service, and opportunities created for delivering true user-led innovation.
Brudney and England (1983)	Brudney and England (1983) present a hierarchical typology, reflecting the distribution of benefits (from the individual to collective). *Individual coproduction*—consists of ‘captured coproduction’, whereby citizens have little choice but to participate in the service and do not control the service agenda (e.g. children may be legally required to attend school). Citizens may also take voluntary behaviours, but these are for their own consumption and benefit (e.g. litter picking). *Group coproduction*—involves voluntary, active participation by a number of citizens with a shared interest (e.g. neighbourhood watch groups). *Collective coproduction*—coproductive activities result in collective goods whose benefits may be enjoyed by the entire community. Collective coproduction programs require direct citizen involvement in service delivery (e.g. voluntary-based fire service), the benefits of which are redistributed across society.	Individual coproduction in climate services is evident when, for example, an individual farm-holder downloads climate information to support strategic planning. Group coproduction is displayed when a network or organisation invests in a climate service; the benefits of which are shared across group members. The Energy Project (EP2) is a good example of this. Sponsored by the UK energy sector, the project will lead to the formation of an energy and climate change industry group who will share knowledge and best practice across the sector (https://www.metoffice.gov.uk/services/climate-services/case-studies/energy). Collective coproduction is apparent in the recent development of the Copernicus Climate Change Service (C3S). The C3S has been informed through stakeholder engagement, the benefits of which will be redistributed across potential users and different sectors through the provision of free services.
Bovaird et al. (2015)	Building on Brudney and England’s (1983) typology, the distinction between individual and collective coproduction is determined by whether the outputs are collectively enjoyed and whether the inputs are collectively supplied (with the potential for hybrid categories). *Private individual coproduction* provides benefits and private value of services to individuals or groups of users. Alford (2002) refers to these as users or clients. *Philanthropic individual coproduction* results in collectively enjoyment. This is typically seen amongst volunteering individuals supporting a public service. *Private collective coproduction* is collectively provided by a group, who in turn gain individual benefits (such as collective self-help groups). *Philanthropic collective coproduction* is collectively provided (i.e. via a group) with the intention to benefit the wider community (such as neighbourhood watch schemes).	Building on the examples above, Bovaird et al. (2015) introduce the notion of *philanthropic* (individual or collective) coproduction, whereby the benefits of coproduction are dispersed beyond the participating individual or organised group and offer a wider societal benefit. In contrast, private (individual or collective) coproduction is apparent in initiatives that benefit individuals only (e.g. an individual farmer) or collectives with a shared interest (e.g. sectoral climate services). For example, the CI4Tea project utilises coproduction processes to provide fit-for-purpose climate information to support climate resilient planning in tea production (http://www.futureclimateafrica.org/news/launch-of-climate-information-for-resilient-tea-production-ci4t-project/). In both cases of private coproduction, it could be argued that the benefits do in fact have the potential to extend to society more broadly; for example, the ability to mitigate and adapt energy supply to changing climates has corresponding benefits to all energy users. Bovaird et al. (2015) similarly acknowledge the difficulty of categorising coproduction into a single category. In this example, coproduction of climate services in the energy sector could qualify as both private and philanthropic collective coproduction.
Mitlin (2008); Watson (2014)	In contrast to the previous examples, other authors have drawn attention to distinct types of coproduction centred on the role of power and transformative change. This has been referred to as *social movement-initiated coproduction*. This is typically discussed in the context of development research in the Global South. Whilst this is beyond the scope of this literature review, it is necessary to acknowledge that such literature has been critical of public administration and service management fields in their instrumental framing of coproduction and neglect of power. This alternative perspective argues that coproduction can be driven by bottom-up initiatives and employed as a political strategy to improve state-society relations and negotiate greater benefits at the local scale (Mitlin 2008). In this context, coproduction is regarded as a strategy for empowering marginalised groups, asserting political influence and effecting change.	Authors have similarly started to call for greater criticality in climate services research, challenging the depoliticised, instrumental nature of coproduction (Goldman et al. 2018). Coproduction processes invariably involve decisions or self-selection biases when it comes to the inclusion/exclusion of service users, which in turn raise implications for the distribution of benefits and prompt social justice debates. Moreover, coproduced climate services have the potential to effect change at the local scale and influence state-society relations. This continues to be a research gap in the climate services field and thus warrants further study.

Beyond designing and implementing more meaningful coproduction in climate services, typologies can also function analytically and draw attention to the underlying assumptions, agendas and practices embedded within climate services, whilst prompting critical reflection into the resulting distribution of benefits and socio-cultural, political and ethical implications of coproduction. Indeed, the need for more criticality has been called for by others (Goldman et al. 2018). Moreover, categorising coproduction can enable meaningful comparisons to qualify the effects of coproduction across different settings (Verschuere et al. 2012). Further research is required to validate and refine an appropriate typology for climate services research; nonetheless, Table 2 provides a useful starting point.

### Motivating coproduction amongst service users

Service-based research has highlighted several (interacting) factors that may influence users’ motivation to engage in coproduction activities, including the type of coproduction, perceived self-efficacy, control beliefs, actor types and trust.

Firstly, there is evidence to suggest that different types of coproduction (Table 2) may appeal to different groups. Comparing across five European countries and focusing on health, community safety and care of the local environment, Bovaird et al. (2015) identify key differences between individual and collective coproduction. For both individual and collective coproduction, self-efficacy (belief in one’s ability to effect change) is a key predictor of participation across all countries. In order of significance, individual coproduction is associated with older citizens, high perceptions of self-efficacy, women and those satisfied with information provided by government, but with low satisfaction in terms of government performance. In contrast, collective coproduction is attributed to high self-efficacy, inactive members of the workforce and increased satisfaction with government in terms of consultation with citizen opinions, with older and more educated citizens least likely to engage in collective coproduction. Variations are also observed between countries and potentially explained by administrative, institutional and social welfare traditions, as well as overall satisfaction in public services.

Related to self-efficacy, the perception of one’s ability to influence the service, referred to as (service) locus of control, has been shown to influence attitudes and adoption of coproduction behaviours (Bradley and Sparks 2002; Fledderus and Honingh 2016). A research by Büttgen et al. (2012) indicates that such control beliefs can be fostered through socialisation activities of service providers, such as methods of communication and training, to help service users/customers to learn and adapt to the values, norms and practices of the organisation. Other strategies that providers may use include simply responding to user needs and enabling users to customise services (van Beuningen et al. 2011; Bovaird et al. 2015). Given the prevalence of self-efficacy and control beliefs in the wider services literature, it is clear that climate services research should examine the extent to which these factors motivate coproduction and, if so, identify pathways through which these may be strengthened.

A further distinction in the literature is made between extrinsic and intrinsic motivation (Fledderus and Honingh 2016). Whereas the former is based on material rewards or punishment and sanctions, the latter is driven by what the individual finds to be interesting, worthwhile or enjoyable. Fledderus and Honingh (ibid) examine the influence of this upon selection biases in the coproduction of public activation services (i.e. services that facilitate the redeployment of jobseekers into the labour market) and observed that those who are highly intrinsically motivated are more likely to engage in such programmes. In this case, there is an individual benefit to be gained; however, others have shown that underlying motivations may differ between different types of actor groups (Alford 2014). According to Alford’s (2002) research into the Australian public sector, clients, users and customers are variably motivated by (i) material rewards (tangible benefits such as money and goods); (ii) sociality incentives (rewards of associating with others); (iii) expressive incentives (intangible rewards related to e.g. sense of goal attainment); (iv) intrinsic rewards (e.g. enhancing sense of self-efficacy); and (v) sanctions (e.g. legal obligations). However, this distinction is not always so clear-cut. For instance, whilst clients signify those that pay directly or even indirectly gain private value from goods or services, Alford observes that clients are not simply motivated by material rewards and sanctions as one might assume, thus suggesting that more complex non-material incentives should be equally understood.

Taking this a step further, van Eijk and Steen (2014) delineate additional types of citizen co-producers. Examining Dutch health services, the authors observe ‘the semi-professional’, ‘the socialiser’, ‘the network professional’ and ‘the aware co-producer’ involved in health care client councils. Each type of co-producer responds to different motivators and has different views on their competence to implement change. Van Eijk and Steen acknowledge the need for further research to examine behavioural differences between these different types of co-producers and whether similar types are observed in other service contexts. Crucially, this research highlights the importance of not thinking about citizens in a singular form. In a similar ilk, climate services research should be cautious to not conceive users as a homogenous group but instead investigate the various roles and motivations driving users’ engagement in the coproduction of climate services.

Finally, trust has proven to be influential in users’ motivation to coproduce. Users need to be convinced of the potential benefits of their participation and service providers’ ability to act upon the users’ contributions (Osborne and Strokosch 2013; Fledderus and Honingh 2016). Supporting this endeavour, the concept of relationship marketing (rooted in service-dominant logic) presents relationships as a valuable resource and stresses the need to create and maintain interactions with customers/users over time (Grönroos 1999: Osbourne et al. 2012), and the benefit of this for fostering trust (Osborne and Strokosch 2013). Thus, it appears that there could be scope for applying relationship marketing to climate services.

This review has highlighted how several (often overlapping) factors influence users’ motivation and willingness to coproduce. Other factors such as capacity, salience and institutional frameworks are also relevant (Verschuere et al. 2012: Alford 2014). Given the range of potential users and public-private spectrum of climate services, it is logical to assume that user participation in coproduction will be motivated by a host of these factors. This highlights the challenge of implementing successfully co-produced services (Fledderus and Honingh 2016). Although further research is warranted, it seems apparent that coproduction in climate services will need to draw from different motivational incentives, recognising that users are a non-homogenous group.

## The influence of ‘servicescapes’

The influential role of service environments (or ‘servicescape’) has been widely studied in service management and services marketing research (Mari and Poggesi 2013), where a service-dominant logic prevails (Section 3.1), but has yet to filter into the climate services domain. As this discussion will demonstrate, servicescapes can influence behavioural intention, value creation and perceived service quality, each of which is highly relevant for the delivery of successful climate services.

The concept of ‘servicescapes’ was first coined in marketing theory by Bitner (1992), inspired by environmental psychology and research examining the influence of physical surroundings upon human behaviour (referred to as the study of atmospherics (Hoffman and Turley 2002)). Servicescapes encapsulate the physical, place-based context of the service encounter, including ambient conditions (e.g. temperature, light), spatial layout and functionality, signs, symbols and artefacts which provide cues about the service and its quality (Nilsson and Ballantyne 2014). Servicescapes provoke cognitive, emotional and physiological responses that influence the individual behaviour of the employee and customer, the quality of their interaction, and customer experiences, expectations and satisfaction. To help articulate this, Bitner uses the example of a restaurant to explain how the furniture and décor help a customer to categorise the establishment (e.g. fast food vs elegant sit-down meal) and trigger emotional arousal (e.g. pleasure responses). Bitner’s seminal work has evolved from focusing on objective, managerially controllable stimuli, to a holistic concept that embraces subjective experiences, shaped through the physical, social, socially symbolic and natural environment (Rosenbaum and Massiah 2011). With advances in computing and the internet, research has expanded into the study of virtual servicescapes or cyberscapes (Williams and Dargel 2004), web atmospherics (Dailey 2004) and e-servicescapes (Hopkins et al. 2009). Regardless of physical or virtual settings, it is argued that servicescapes influence the meanings that customers associate with value propositions, their expectations and satisfaction (Nilsson and Ballantyne 2014, p. 377).

Climate services must similarly meet users’ expectations and needs; therefore, the servicescapes through which providers and users interact is equally relevant. These servicescapes (typically virtual) can be strategically managed to achieve the goals of both service providers and users. Managed servicescapes can assist customers/employees in their activities, help differentiate the intended market and convey distinctiveness from competitors (Bitner 1992). For climate services, careful management of the servicescape (such as website design and navigation options) could promote desired behaviours (e.g. downloaded or purchased information) and improve user satisfaction. Therefore, as argued by Bitner (ibid), we assert the value of designing and implementing servicescape strategies in climate services.

This requires further research into the key components of climate service ‘servicescapes’. For example, Williams and Dargel (2004) document the influence of website content and design (e.g. vividness, interactivity and sense of control) upon browser responses (e.g. prolonged usage, intention to return and recommend to others) in the context of e-businesses. Just as physical settings may be imbued with cues (signs and symbols), these also exist virtually and can alter browsers’ perceptions (e.g. trustworthiness). Tailoring virtual servicescapes to optimise user experience requires an understanding of user requirements; however, this is often challenged by the range of users, with varied competencies and interests. The same observation is true of climate services (Bruno Soares et al. 2017). A useful strategy is to impart control onto the user to actively select the content and flow of information, essentially customising their service experience (Williams and Dargel 2004). In turn, this may empower users as co-creators of the service, build self-efficacy and motivate coproduction (Osborne and Strokosch 2013: Section 3).

Another important aspect of virtual servicescapes is the creation of spaces for customer-to-customer interaction, such as online forums. As demonstrated by Blasco-Arcas et al. (2014), this can help forge favourable relationships between the customer and the firm, as well as increasing customer interest in coproduction. In this sense, Blasco-Arcas et al. (2014) describe virtual engagement platforms as touch points for interaction and co-created value. However, there is also a need to remain cognisant of the socially symbolic meaning ascribed to signs and symbols in digital settings (‘socially symbolic servicescape’), which can affect the inclusion/exclusion of certain users (Rosenbaum 2005). For example, the presentation and accessibility of climate information through online platforms of climate services may prove more amenable to certain groups (e.g. so-called expert users) over others. Indeed, several experiential barriers are identified by Hewitson et al. (2017) in relation to assumed familiarity with terminology, navigation, clarity of information and multiple choice.

This review demonstrates the importance of considering users’ experience of the service environment itself. Few authors have acknowledged this in climate services; for instance, Hewitson et al. (2017) acknowledge that user experience is paramount to the added value of climate information websites. Moving forward, we call for a new avenue of research into the servicescape of climate services. This research should consider the various pathways through which the service environment may be tailored to user needs and altered accordingly to improve access to, and experience of, climate services amongst different user groups. The web-based interface need not be a passive form of user engagement (Hewitt et al. 2017). It is vital that such research meaningfully engages with (potential) users in the coproduction of servicescapes (Buontempo et al. 2018). Moreover, such research would benefit from consultation with other disciplines, such as web design (Christel et al. 2018). This endeavour should be pursued for different types of climate services, servicing different users and uses, in order to identify shared and unique features of the servicescape to inform appropriate servicescape strategies.

## Lessons for climate services

Given the rapid expansion of climate services, this article presents a timely analysis of the extent to which climate services scholarship can translate lessons from other service contexts, drawing from the long legacy of public administration, service management and marketing literature. Focusing on themes of *coproduction* and *servicescapes*, this research has identified the following:

- Coproduction can be approached from a goods-dominant logic and seen as an ‘add on’ component (public administration and new public management), or through a service-dominant logic where it is seen as integral to the service (service management and service marketing). Climate services have arguably tended towards a goods-dominant logic, focusing on the supply of climate information products and coproduction that is steered and controlled by service providers, whilst neglecting the service experience and intrinsic role of service users. We therefore suggest that climate services could benefit from embracing a more service-dominant culture that recognises the importance of users’ subjective experiences and empowers users as co-producers of value.
- Numerous typologies of coproduction exist within the broader services literature that have yet to filter into the climate services field. Applying these typologies to climate services could provide a valuable lens for future research, from documenting current modes of coproduction witnessed in climate services, to acting as analytical frameworks and drawing attention to the different underlying assumptions, agendas and practices embedded within. In turn, this could prompt critical reflection into the resulting distribution of benefits and socio-cultural, political and ethical implications of coproducing climate services.
- Numerous factors influence service users’ motivations to coproduce services (self-efficacy, perceived control, actor-type and trust). Further research is required to examine these factors and their potential variation with different modes of coproduction. Nonetheless, it seems apparent that coproduction in climate services will need to draw from different motivational incentives, recognising that users are a non-homogenous group.
- Users encounter services through physical and/or virtual servicescapes, which influence their expectations, perceptions (e.g. trust, service quality) and behaviours. We call for a new avenue of research into servicescapes, to consider the various pathways through which the service environment may be tailored to user needs and altered accordingly to improve both access to, and experience of, climate services amongst different user groups. It is vital that such research meaningfully engages with users and relevant disciplines (e.g. web design). In turn, such research could inform the development of appropriate servicescape strategies.

This article has drawn examples from both public and private services, recognising that climate services are situated across this spectrum, with a growing trajectory towards marketisation, similarly witnessed in weather services (Randalls 2010; Webber and Donner 2017). However, it is necessary to acknowledge that the commercialisation of climate services is strongly contested with arguments that this could restrict science to commercial applications, undermine data quality (Randalls 2010), as well as reinforce social inequalities (Webber and Donner 2017). This has led to calls for greater criticality towards the ethical considerations of climate services and their coproduction (de la Tozier and Daly 2017: Goldman et al. 2018). Although this article sought to highlight the potential benefits of learning from other service contexts (including the private sector), we equally wish to emphasise the need for scholars to, at the very least, remain critical of the language and semantics describing climate services, and acknowledge this growing debate amongst the climate services community.

Nonetheless, this analysis has highlighted some interesting knowledge gaps for climate services research to address, pertaining to coproduction and servicescapes. These observations should be understood within the boundaries of the sampled literature and corresponding biases; therefore, further research is required to examine the extent to which these findings and lessons are applicable across different types of climate services, scales and contexts. Given that these themes were selected as starting points only, we encourage others to similarly mine the repository of service-based research. Involving experts in service management, marketing and public administration could support this endeavour and offer an alternative perspective on climate services, as well as opportunities for future interdisciplinary collaborations. This is a necessary step in broadening the conversation around climate services and advancing both scholarship and operational delivery.

## Footnotes

1 Certain subject areas were excluded from the search, including medicine; materials science; biochemistry, genetics and molecular biology; mathematics; pharmacology, toxicology and pharmaceutics; physics and astronomy; chemical engineering; immunology and microbiology; neuroscience; dentistry; chemistry; veterinary; and undefined subject areas. Date of search 27/07/2018.
